# Denture microbiome shift and changes of salivary inflammatory markers following insertion of 3D printed removable partial PMMA denture: a pilot study

**DOI:** 10.1186/s12903-024-05012-z

**Published:** 2024-10-14

**Authors:** Norlela Yacob, Syarida Hasnur Safii, Norasmatul Akma Ahmad, Norsiah Yunus, Fathilah Abdul Razak

**Affiliations:** 1 Department of Restorative Dentistry, Faculty of Dentistry, Universiti Malaya, Kuala Lumpur, 50603 Malaysia; 2 Department of Conservative Dentistry & Prosthodontics, Faculty of Dentistry, Universiti Sains Islam Malaysia, Nilai, Negeri Sembilan Malaysia; 3 Department of Oral and Craniofacial Sciences, Faculty of Dentistry, Universiti Malaya, Kuala Lumpur, 50603 Malaysia; 4 School of Dentistry, Management & Science University (MSU), Shah Alam, 40100 Malaysia

**Keywords:** Denture, Microbiome, Oral biofilm, 3D printing, Resin

## Abstract

**Background:**

The aim of this study was to investigate the microbiome shift of denture biofilm formation, the incidence of Candida and changes of salivary inflammatory markers following insertion of 3-dimensional printed PMMA denture.

**Methods:**

This was a pilot study of 6 patients provided with 3D printed (test group) or conventionally heat-cured (control group) removable partial acrylic dentures followed up for 6 weeks. 3 denture swabs were collected at week 1, 3 and 6; and saliva samples were collected at baseline, week 1, 3 and 6 following denture insertion. Microbial DNA was isolated, and the 16S rRNA gene was amplified and sequenced to assess the denture microbiota. The presence of Candida was determined using PCR assay. The levels of salivary biomarkers lactoferrin (LTF) and histatin (HTN) were determined using ELISA.

**Results:**

Denture microbiome of both groups exhibited similar microbial compositions at weeks 1, 3 and 6. However, the percentage of *Streptococcus* in the test group was significantly lower at week 1 (*p* < 0.05). Greater bacterial diversity was detected in the test group (*p* < 0.05; pairwise Wilcoxon) after 6 weeks. The presence of Candida was only detected in one sample of the 3D printed denture (test group) after 6 weeks. The level of LTF biomarkers increased in both groups after 6 weeks, however, the level of HTN increased only in the control group.

**Conclusions:**

Denture microbiome of both groups demonstrated similar microbial compositions. After 6 weeks, 3D printed denture demonstrated higher diversity with delayed microbiome shift compared to conventional heat cure dentures. 3D printed denture may be a viable alternative to conventional denture and it has a potential to delay microbial dysbiosis.

**Clinical Relevance:**

Among wearers, 3D printed denture can potentially reduce the risk of denture stomatitis. A greater diversity may delay or slow the transition of denture microbiome to dysbiosis.

**Supplementary Information:**

The online version contains supplementary material available at 10.1186/s12903-024-05012-z.

## Introduction

Dentures are effective in replacing missing teeth and improving patients’ quality of life. However, denture-wearing may lead to development of mucosal infections especially in susceptible geriatric patients [1]. Biofilm that forms on dentures can serve as reservoirs for various pathogens that affect oral and systemic diseases [2]. Among mucosal infections and conditions, denture stomatitis (DS) is the most common inflammatory condition that affects mucosa of denture-bearing areas [2].

Microbial composition of biofilm on dentures closely resembles that of natural teeth [4], but the types and proportions of microorganisms vary depending on factors such as presence of natural teeth or edentulism [5]. Swabs from denture fitting surfaces revealed that the dominant bacteria were *Streptococcus* spp. (*S. mutans*,* S. mitis*,* S. salivarius* and *S. sanguinis*), *Actinomyces* spp. (*A. israelii*,* A. nueslundii*,* A. odontolytic*), *Lactobacillus* spp. and *Veillonella* spp. [6, 7]. Apart from with these bacteria, *Candida* spp. (*C. krusei* and *C. glabrata)* was present in low percentage [8]. Candida is more prevalent on dentures than on tooth surfaces [9, 10], especially when oral hygiene is poor or if denture stomatitis is present [11].

Next-generation sequencing (NGS) technology has enhanced our understanding of microbiome. Researchers classify the compositions into three groups: health-associated species, core species, and disease-associated species [12]. Ideally, there should be more health-associated than disease-associated species to prevent disease from developing. A balanced and harmonious relationship exists between the oral microbiome and host factors and is known as symbiosis [7]. Under healthy conditions, a symbiotic state elicits a minimal inflammatory response that does not lead to tissue destruction, while the microbiome environment is beneficial to both microbes-to-microbes and microbes-to-host [17, 18]. When the symbiotic state is disrupted, the composition of bacterial community within the biofilm ecosystem changes, leading to decrease diversity and unbalanced environment which is referred to as dysbiosis [4, 19].

In relation to denture stomatitis (DS), a few studies have been conducted to assess the microbiome of denture [4, 5, 13]. Bacilli and Actinobacteria are the dominant phyla on denture surfaces [5]. The abundance of Actinobacteria and Bacilli is approximately three times greater in healthy mucosa than in DS patients [5]. At genus level, the bacterial compositions in denture biofilms are similar between patients with DS and those with healthy conditions, except for Streptococci (*S. gordonii*,* S. sanguinis*, and *S. australis*) which is found in higher level in the latter group [13]. Furthermore, a more diverse bacterial composition was observed in denture biofilms of healthy patients [14].

There is strong evidence of interaction and competition between bacteria and Candida species to adhere to the denture surface [15]. Candida species interacts synergistically with *Streptococcus* and *Staphylococcus* species, forming a robust bond with the biofilm and extracellular polymeric substance (EPS) matrix [16]. This leads to resilient biofilms, which are difficult to remove [14]. In denture stomatitis, the level of *Candida* sp. positively correlated with *Lactobacillus* sp. and negatively correlated with the *Fusobacteria* sp [5]. Furthermore, Candida has been detected in both healthy and stomatitis conditions. Certain bacteria, such as *Streptococcus* sp., *Pseudomonas* sp., and *Stenotrophomonas* sp., have been found to influence the presence of Candida in DS patients [4, 13].

The impact of the denture microbiome on 3D printed denture resin in oral environment is not well understood. A few studies have shown that adherence of Candida is notably higher on 3D printed dentures than on conventional heat cure (HC) dentures [20, 21]. In contrast, other research has shown that adherence of Candida was significantly reduced when Candida was examined together with mixed microorganisms, such as *Streptococcus*,* Staphylococcus*,* Corynebacterium* and *Porphyromonas* [22] which suggests that the results may be influenced by research settings. Even though similar microbiome develops on a denture surface, the initial adherence of early colonisers appears to differ slightly based on the properties of the materials used [23, 24].

This study aimed to investigate the microbiome shift including incidence of Candida and changes of salivary inflammatory markers (LTF and HTN) following insertion of 3D printed PMMA denture and compared to conventional HC PMMA denture resin at week 1, week 3 and week 6.

## Methodology

### Patient recruitment

This was a pilot study of 6 patients provided with (i) 3D printed (test group) or (ii) conventionally heat-cured (control group) removable partial acrylic dentures from two centers: (i) the Postgraduate Dental Clinic (Prosthodontic Clinic), Universiti Malaya (UM), and (ii) the Prosthodontic Specialist Clinic, Universiti Sains Islam Malaysia (USIM). Medical ethics approval to conduct this study was obtained from the Medical Ethics Committee, Faculty of Dentistry, Universiti Malaya (reference No: DF RD2104/0013 (P)) and in accordance with the amended World Medical Association Declaration of Helsinki 2013.

The patients did not have any active oral disease, non-smoker and non-alcohol consumer, not on pregnancy and contraceptive pill, and had not received antimicrobial or antifungal treatment for the past 6 months. The cause of tooth loss either from periodontal disease or caries was accepted. A prosthodontist was involved in collecting sociodemographic data, denture history, dental status; assessment of mucosa, bleeding on probing (BOP) score, clinical examination, provision of prosthesis and assessment of mucosal inflammation of the denture-bearing area (Y/N) using Newtons’ Classification.

### Denture fabrication

The design of the dentures was standardised according to Kennedy III Classification, a minimum of two retention components (clasp), a support component (rest) where applicable, and a major connector (palatal strap/plate) covering two-thirds of the posterior palatal area. The workflow for denture fabrication was adapted [25] and is presented in Supplementary 1. The denture design using CAD software program (3Shape Dental Designer, USA) was transferred to the printing software (3DSprint, USA). The nesting procedure with build orientation was set using the optimisation features for Surface Quality available in the software. Once the denture was printed with DLP printer (NextDent 5100, USA) using denture base resin Denture 3+, translucent shade (NextDent, USA), and the resin for teeth was Crown & Bridge Micro Filled Hybrid (C&B MFH, NextDent, USA). Subsequently, it was cleaned for 3 min in iso propyl alcohol (99.9%) and washed again for 2 min using ultrasonic bath, and post polymerised for 30 min with blue ultraviolet-A (UV-A) at 315–400 nm wavelength in a light cure box (LC-3DPrint Box; Vertex-Dental BV). The denture was fitted into patient’s mouth for try-in. Once satisfied, 0.7 mm stainless steel wire clasps and rest were added where applicable and further processed using laboratory self-cure resin (Hugh Dent, Shangdong) and polishing. For conventional heat-cure denture base resin (PMMA Huge, China), a similar initial procedure was performed with a heat compression technique at 74 °C for 8 h followed by 100 °C for a 1-hour cycle using denture base resin. Throughout the study period, every patient was instructed to wear the denture continuously and to remove it only for cleaning purpose. During cleaning, the denture was rinsed under running tap water, without the use of any brushes and denture cleanser.

### Clinical sample collection

A total of 18 samples were obtained from the denture surface at weeks 1, 3 and 6 from the test (*n* = 3) and control groups (*n* = 3). All patients were asked to wear their denture for at least 3 h and refrain from eating and drinking for at least 1 h prior to every sample collection. Immediately after the denture was removed, a sterile cotton swab was rubbed on the two-thirds of denture fitting surface for 15 s [14]. The swab was then transferred to a 1.5 mL microcentrifuge tube containing 300 µL of DNA/RNA shield buffer (DNA/RNA Shield™, Zymo Research, Irvine, CA, USA). All samples were stored in a -80 °C freezer until further analysis.

At the same time, 5 ml of unstimulated saliva was collected between 9:00 am and 11:30 am on the day of denture insertion (baseline) and at review follow-ups. A trained dentist supervised the collection of saliva from each subject. Subject spitted out the saliva into a chilled polypropylene tube at 30 s intervals for a period of 5–20 min. The saliva samples were centrifuged at 1,000 × g for 20 min at 4 °C. The aliquot supernatant was collected, and the samples were stored in a -40 °C freezer until analysis. Both sample collections were processed by a microbiologist who was blinded to the treatment allocation.

### DNA extraction

Bacterial genomic DNA was extracted from the denture swabs using a QIAamp DNA Mini Kit (Qiagen, Valencia, CA, USA). The quantity and purity of DNA were determined using a spectrophotometer (Implen NanoPhotometer^®^ N60/N50, Portugal) and a dsDNA Quantification Kit (iQuant™ Broad Range, ABPBioscience, USA) using fluorometric quantification according to the manufacturer’s instructions. All samples were subjected to 16S rRNA gene sequencing. Amplicon sequence variant (ASV) generation via the DADA2 workflow was used [26].

### Next-generation sequencing

The quality of the libraries was measured using an Agilent Bioanalyzer 2100 System with an Agilent DNA 1000 Kit, and fluorometric quantification was performed with Helixyte Green™ quantification reagent. The amplified products were cleaned for the second time using AMPure XP beads (Beckman Coulter, Indianapolis) for the final library before quantification. The libraries were normalised and pooled according to the protocol recommended by Illumina System (Illumina Inc., San Diego, USA), and sequencing was performed with MiSeq platform using 300 PE.

### Data analysis and taxonomic composition analysis

The 16S rRNA sequence from the microbial taxonomic composition analysis was clustered into bacterial operational taxonomic units (OTUs) and computed with Quantitative Insights into Microbial Ecology (QIIME 2). OTUs were then interpreted and compared with representative sequences from the Human Oral Microbial Database [27].

### Detection of Candida

The presence of Candida in the denture samples was evaluated through PCR amplification using the following primers: the universal forward primer FP ITS1 (5’ CTT GTT ATT TAG AGG AAG TAA 3’) and the reverse primer ITS2 (5’ GCT GCG TCC TTC ATC ATGC 3’) (Shi et al., 2016) with PCR Master Mix (1st BASE, Singapore). The PCR cycle conditions were as follows: 94 °C for 11 min; 35 cycles of 94 °C for 30 s, 50 °C for 30 s and 72 °C for 30 s; and a final 30-min extension at 72 °C and a 4 °C hold time in a thermocycler (T100, Thermal Cycler, Bio-Rad, USA). Electrophoresis through 1.5% agarose gel (Promega, USA) was carried out to detect the presence of Candida using a Bio-Rad PowerPac apparatus, and *Candida* spp. were then transferred to a UV illuminator Gel Doc™ eZ Imager (Bio-Rad, USA) for electrophoresis. The presence of DNA band was detected using the Image Lab™ software program (Bio-Rad; USA).

### Saliva biomarker assessment

The aliquoted supernatant samples were thawed prior to ELISA analysis (ABclonal, China) for salivary histatin (HTN) and lactoferrin (LTF) biomarkers. The assay was performed according to manufacturer instruction. The optical density (OD) was recorded using a microplate reader (Uquant, Biotek). The data was processed using software (Gen5 ELISA) to get the corrected absorbance value (nm). Based on the graph generated from the standard curve plot, the LTF and HTN concentrations were analysed. The assay was performed in triplicate.

### Data analysis

Metagenomic data was analysed as alpha and beta diversity based on the OTUs. Kruskal‒Wallis analysis was used to compare the intra- and intergroup differences of alpha diversity. For beta diversity, the significant difference in community structure was confirmed using the Adonis one-way permutational multivariate analysis of variance (PERMANOVA) test. Two-way repeated measures (RM) ANOVA was used to examine the difference of mean concentrations of LTF and HTN biomarkers of the 2 groups across the time intervals.

## Results

### Demographic and clinical outcomes

The demographic data, history of wearing dentures, current oral hygiene status and number of teeth are tabulated in Table 1. Mean age of the patients was 50.33 (±9.5) years, and mean number of teeth present was 17.5 (±4.3). None of the 6 patients had denture stomatitis lesions at the follow-up visits: week 1, week 3, or week 6.

**Table 1:** Demographic characteristics, denture experience, number of teeth and oral hygiene status of the patients

Subject ID	Sex	Age	Denture experience			Number of teeth present	Oral hygiene
			Previous denture experience	Denture wore at present	Age of present denture		
3D1	F	51	2 dentures	P/-	< 2 months	22	Fair
3D2	M	50	1 denture	P/-	> 1 year	16	Fair
3D3	F	33	1 denture	P/-	< 1 year	21	Good
HC1	M	51	None	NA	NA	10	Fair
HC2	M	64	None	NA	NA	19	Good
HC3	F	53	1 denture	P/-	> 5 years	17	Fair

^3D: 3D printed, HC: heat cure, M: male, F: female, NA: not applicable^

### Illumina sequencing output

All the samples (*n* = 18) from the test and control groups were processed. A total of 221,454 sequences containing the V3-V4 region were investigated, and after processing and trimming of the disqualified sequences, the total number of final reads was 161,981. Each sequence represents a biological entity or a species. Similar sequences (> 97% similarity) were clustered together and defined as operational taxonomic units (OTUs). The OTUs were subsampled to the lowest read count (77,694) to normalise the results between samples (supplementary files: suppl_2, suppl_3).

### Biofilm formation at different time intervals

**Fig. 1:**
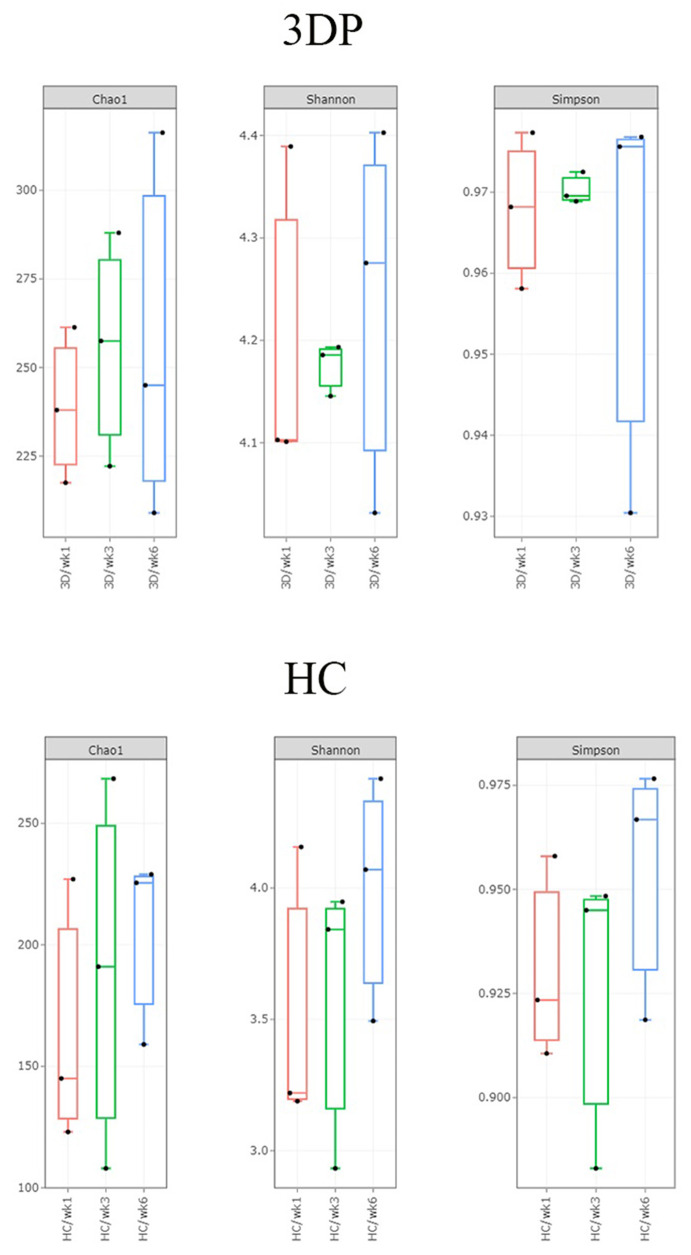
Box and whisker plots showing the number of unique bacterial species (Chao1 index, Shannon Simpson) in the denture biofilms of the 3D printed (test) and HC (control) groups from week 1 to week 6 after denture insertion

**Fig. 2:**
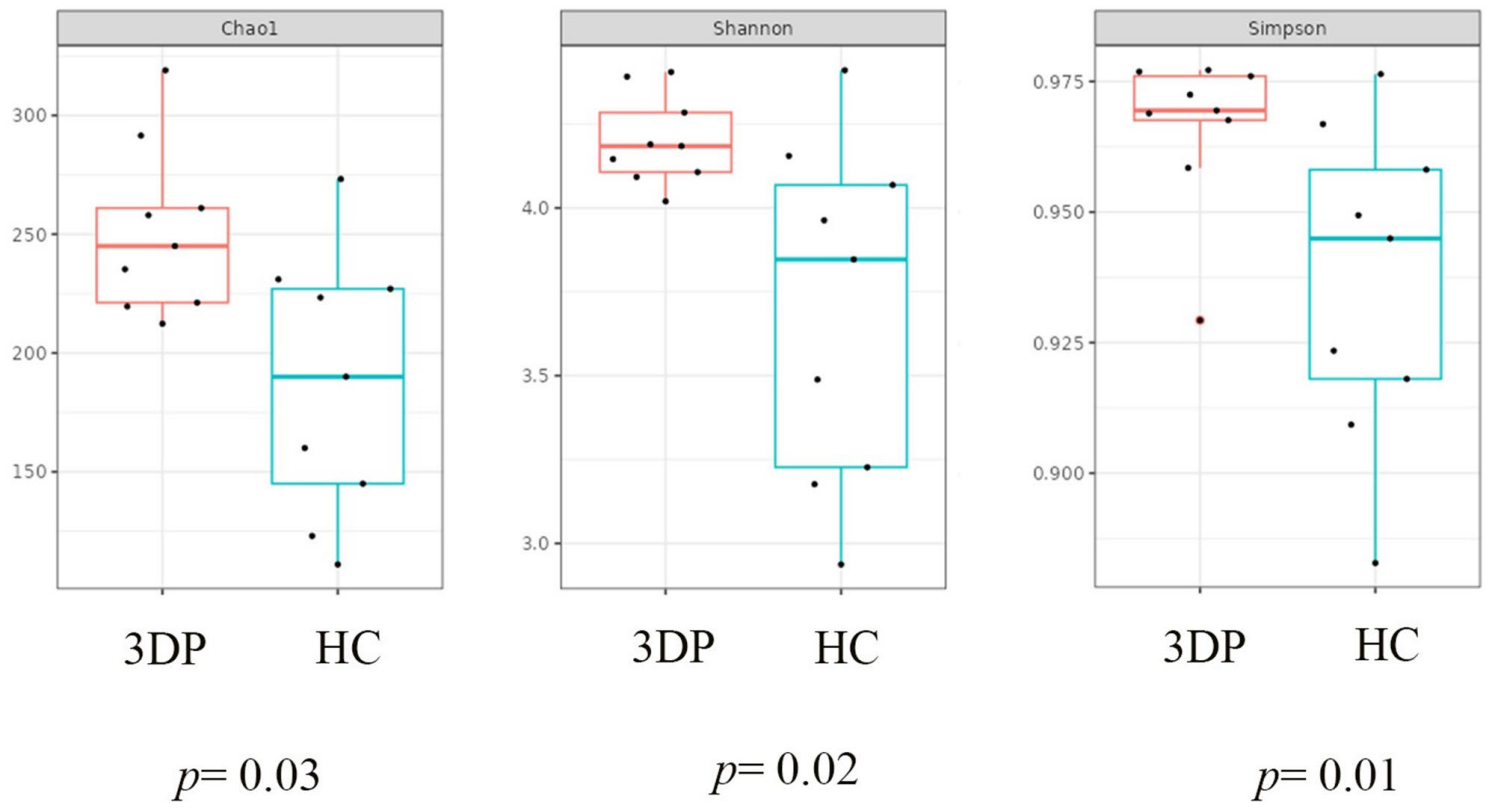
Box and whisker plots showing the overall composition of unique bacterial species (Chao1 index, Shannon Simpson) in the denture biofilms of the 3D printed (test) and HC (control)

Figures 1 and 2 depicts alpha diversity of the bacterial compositions based on Chao1 index (species richness), Shannon index and Simpson index at week 1, week 3 and week 6. Generally, there was a significantly higher number of OTUs in the 3D printed group than the HC group (Chao1, *p* = 0.03). The 3D printed group exhibited a more diverse microbial community than the conventional HC group (Simpson, *p* = 0.01) (pairwise Wilcoxon test).

**Fig. 3:**
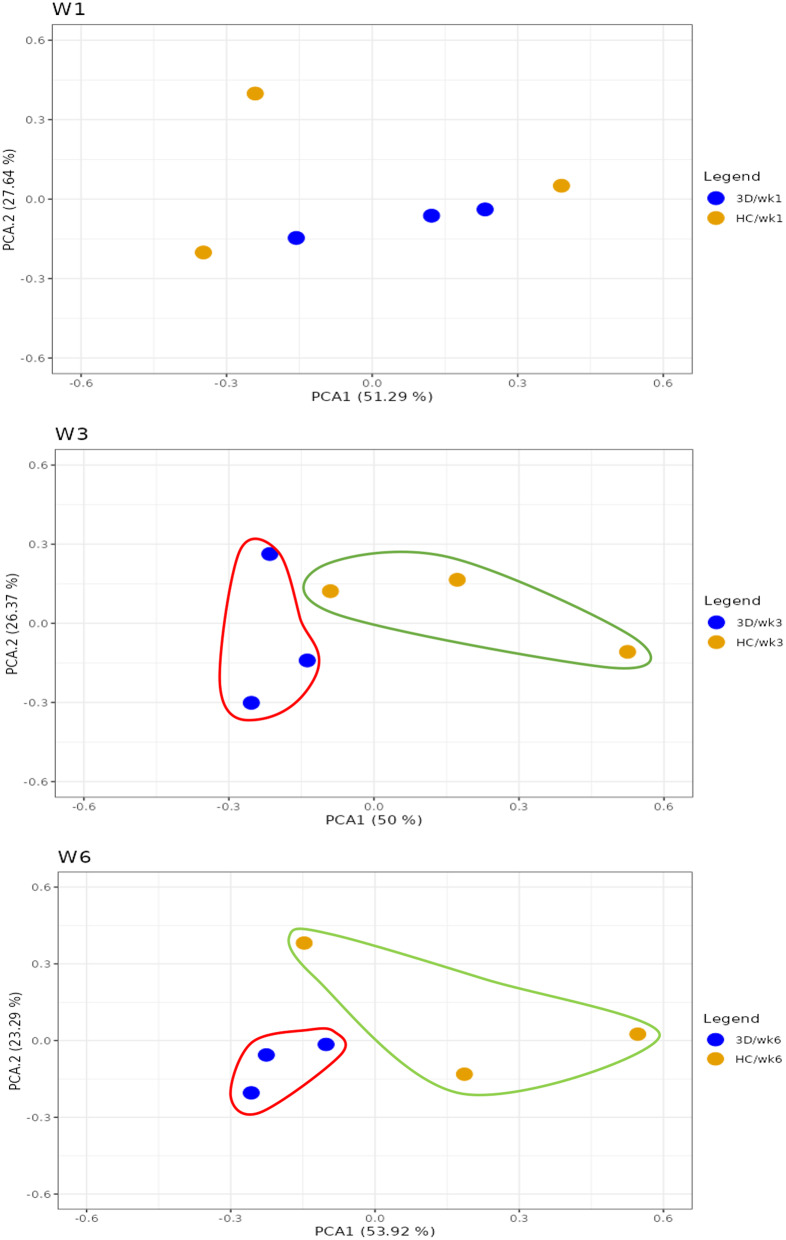
Scatter plot of principal coordinate analysis (PCoA) for beta diversity at various time intervals

Figure 3 shows the scatterplot of beta diversity (principal coordinate analysis [PCoA]) of the bacterial community-colonising dentures (3D printed and HC groups) at different time intervals following denture insertion (week 1, week 3 and week 6). A shift in relative abundance of the bacterial community from 1 week to 6 weeks post denture insertion was observed in both groups. Although there was a trend towards separation, the difference was not statistically significant (*p* > 0.05, ANOSIM).

### Relative abundance

**Fig. 4:**
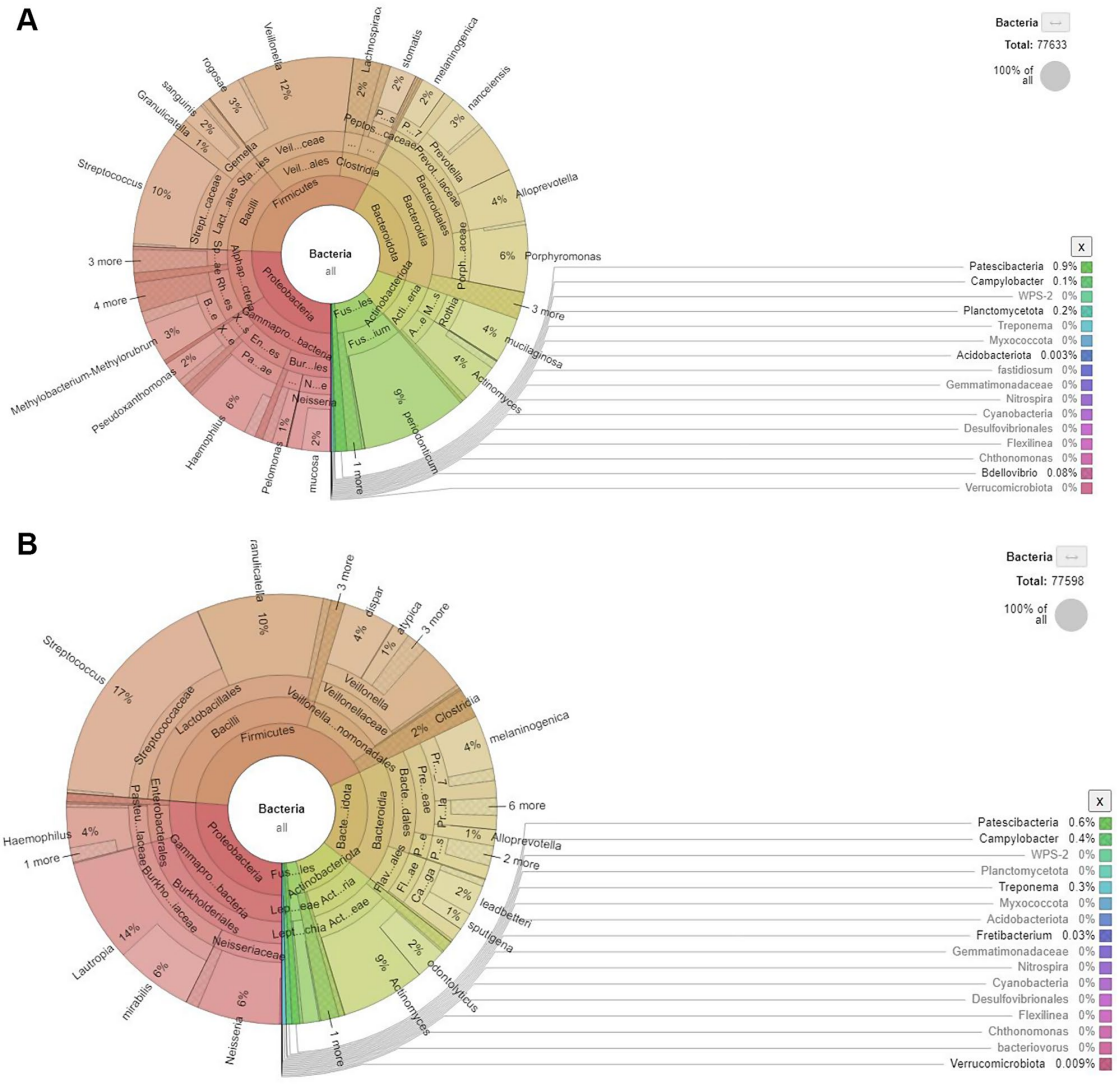
The genera found in 3D printed denture (3DP) and conventional denture (HC) after 6 weeks of denture insertion

The top ten relative abundances are shown in the supplementary file (see Suppl_4). Table 2 shows relative abundance of phyla and genera that were detected only in the 3D printed group after 6 weeks which indicates presence of more diverse microbial community in the test group. The phyla Proteobacteria included Sphingopyis, Methylobacterium-Methylorubrum, Pseudoxanthomonas, and Paelomonas. However, some of them had very low percentage abundances. Figure 4 shows the genera that was found in both test and control groups.

**Table 2:** Several genera were discovered on the 3D printed denture after six weeks but not on the conventional HC denture

Phyla	Genera	Relative abundance %
*Proteobacteria*	Methylobacterium-methylorubrum	3%
	Pseudoxanthomonas	2%
	Paelomonas	1%
	Sphingopyxis	< 1%
	Phenylobacterium	< 1%
	Brevundimonas	< 1%
	Bosae	< 1%
	Afipia	< 1%
	Mesorhibozium	< 1%
	Ensifer	< 1%
*Actinobacteria*	Gordonia	< 1%
*Bacteriodota*	Elizabethkingia	< 1%

### Detection of Candida on denture fitting surfaces

Only one sample from the test group showed presence of Candida 6 weeks after denture insertion (see Table 3). Clinical assessment revealed that none of the patients developed denture stomatitis after 6 weeks of denture wearing.

**Table 3:** Detection of Candida using PCR in all patients

Type of Denture	Sample ID	Time interval	*Candida* sp.
3D printed denture	3D1	Week 1	-
		Week 3	-
		Week 6	+
	3D2	Week 1	-
		Week 3	-
		Week 6	-
	3D3	Week 1	-
		Week 3	-
		Week 6	-
HC group	HC1	Week 1	-
		Week 3	-
		Week 6	-
	HC2	Week 1	-
		Week 3	-
		Week 6	-
	HC3	Week 1	-
		Week 3	-
		Week 6	-

### Saliva biomarkers

**Fig. 5:**
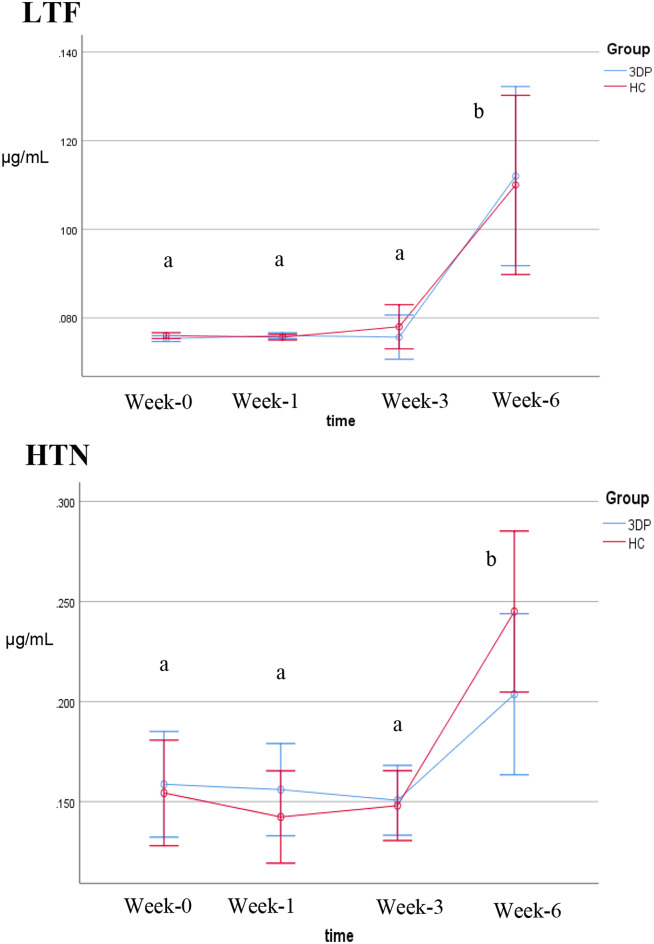
The level of LTF and HTN biomarkers at different times interval

The mean concentrations of LTF and HTN at different time intervals are presented in Fig. 5. There was no significant difference from baseline to week 1 or from week 1 to week 3 (Bonferroni, *p* > 0.05). Interestingly, a significant increase in HTN biomarkers was detected only in conventional HC dentures (*p* < 0.05, Wilcoxon rank test). Further analysis on the comparison between baseline and week 6, showed significant changes on LTF and HTN except on test group in HTN biomarker (*p* > 0.05, paired t-test). Comparison between test and control group showed significant changes on HTN biomarker at week 6 (*p* < 0.05, Wilcoxon rank). The details be found in the supplementary material (see Suppl_5).

## Discussion

This study aimed to evaluate the shift of bacterial composition on the denture fitting surface of 3D printed DBR and the inflammatory changes observed for 6 weeks post denture insertion. Research on denture microbiome mainly investigated candidal infections, specifically examining microorganisms associated with both healthy mucosa and denture stomatitis [4, 5, 13]. The present study focused on the formation of biofilms on new denture fitting surfaces fabricated using 3D printed technology compared to the conventional PMMA resin in partially dentate healthy patients.

The results showed that the species richness and evenness of the denture biofilm were higher in the 3D printed denture group than in the conventional denture group, of which all the patients had healthy mucosa. Several researchers have suggested that surface roughness directly influences microbial adherence on denture surfaces [28–30]. However, the present study showed that even when smoother 3D printed denture surface fabricated with optimised build orientation in 3D printing was used, microbial adherence on the 3D printed resin was still higher than the conventional HC denture base resin, but with diverse community. The chemical composition of different denture resins or brands influences the surface energy of the resin [31, 32]. In addition, build orientation influences surface roughness [33, 34], thus increasing the potential adherence of microorganisms to 3D printed resin [33–35]. All of these factors play a major role in the hydrophobicity of the denture base resin. 3D-printed denture base resin with high hydrophobicity increases plaque accumulation, staining and water sorption more than HC PMMA [32, 36, 37]. Although a smooth surface reduces hydrophobicity, it does not significantly affect microbial adhesion [38]. This suggests that even though 3D printed dentures have a higher affinity for microbial adherence, having healthy microbial communities is important for preventing disease.

The bacterial compositions at the initial stage of biofilm formation in the test and control groups were largely similar in the first week of wearing the denture. Both groups exhibited similar bacterial genera, such as *Streptococcus*,* Prevotella_7*,* Veillonella*,* Klebsiella*,* Actinomyces*,* Haemophilus*,* Rothia*,* Porphyromonas*,* Bosea*,* Neisseria* and others. However, the percentage of mean relative abundance of these bacteria changed after 3 and 6 weeks. For instance, the mean relative abundance of *Streptococcus* in the conventional HC resin group exhibited a statistically significant one-fold increase compared to the 3D printed DBR group during the first week (*p* < 0.05). Although both resins were composed of PMMA, the fabrication methods such as 3D printing, conventional heat cure or milling can also influence the initial colonisation of bacteria [39]. Similarly, Shim et al. (2020) reported that smoother 3D printed surfaces had less adherence of early colonisers. This indicates that different surface materials may influence the adherence of early colonisers.

Findings from the present study showed that the relative abundance of *Streptococcus* and unclassified bacteria were predominant in both denture base resins. In agreement to this, de Castro [40] reported a reduction in the relative abundance of *Streptococcus* in a biofilm after 7 days compared to 24 h, whereas the unclassified bacteria increased after 7 days. Unclassified bacteria refer to new or uncharacterised microorganisms from the sequence used. Unclassified bacteria in the denture biofilm presented with the highest percentage in relative abundance, especially on the 3D printed dentures after 3 and 6 weeks. Further investigation is warranted to characterise unclassified bacteria to improve our understanding of the compositional dynamics of the microbiome in biofilm formation.

The number of different microbial species increased from week 1 to week 3 and again from week 3 to week 6 which indicates that the denture biofilm colonies expanded over time. As biofilm matures, some bacteria will detach from the colony and resettle elsewhere to form new biofilm [41]. Similar findings were reported by Teles and colleagues (2012) whereby they used different methods to count DNA probes and found that mature denture biofilms had similar total number of bacteria but different proportions of species [42]. If the biofilm continues undisturbed, the risk of mucosal inflammation may arise with shift microbiome.

Although the difference in beta diversity between the test and control groups was not significant, there was a noticeable trend in the relative abundance of bacterial communities from week 1 to week 3 and week 3 to week 6 in both groups. Studies have shown that bacterial shift occurs when the environment changes [43, 44]. One study revealed that there was an increase in bacterial biomass and a shift in the microbial community from Gram-positive cocci and rods to Gram-negative bacteria after 3–6 weeks [45, 46]. However, the findings from the present study showed that there were no clinical signs of mucosal inflammation at 6 weeks post denture insertion, indicating no development of denture stomatitis. This could be attributable to the transition of bacteria from the adherence stage to the biofilm maturation stage that did not involve colonization of microorganisms associated with denture stomatitis such as Candida, *Lactobacillus*, *Pseudomonas* and *Staphylococcus.*

After 6 weeks, both denture groups had an increase in microbial community and greater diversity in bacterial composition, indicating maturation of the denture biofilm into a symbiotic state [3, 12]. Despite this, the bacterial composition in the biofilm remained balance with no signs of mucosal inflammation or disease. Absence of stomatitis lesions among the subjects suggests that the diverse microbial composition has not become pathogenic. Studies have shown that stomatitis typically involves changes in microbial composition of certain microorganisms such as *S. gordonii*,* S. sanguinis*,* S. australis*,* F. nucleatum* [12] and *Pseudomonas fuorescens* [3], which are significantly higher in disease compared to health.

By 6 weeks, several genera disappeared from the biofilm on conventional denture but they were present on 3D printed dentures, which pose a risk of developing mucosal infection on the conventional denture. Our results revealed that the phyla Proteobacteria included Sphingopyis, Methylobacterium-Methylorubrum, Pseudoxanthomonas, and Paelomonas. Other study has linked Sphingopyis to oral cancer lesions, however their abundance is greater in healthy patients compared to cancer patient [47]. It may act as predictor in oral cancer lesion. Similar to Pseudoxanthomonas, it is related tonsil hypertrophy [48], but the role of these Proteobacteria in disease are not well documented yet. Their effects on oral mucosa in contact with 3D printed resins which is a new technology warrants further investigation.

At phylum level, the relative abundances of Firmicutes, Proteobacteria, Bacteroides, Actinobacteria and Fusobacteria were similar in both denture types at week 1. Similar results were found in healthy individuals [12, 42]. The top five relative abundance of bacteria at genus level at week 1 and week 3 in both denture groups were Streptococcus, Veilonella, Klebsiella, Haemophilus and Neisseria. At week 6, the pattern was different whereby Streptococcus and Actinomyces came second in the relative abundance. Similarly, Morse and his colleagues [3] reported that the abundance of Actinomyces was six times greater in healthy mucosa. One study reported that Actinomyces rather than Streptococcus was at the top of the list for genera associated with healthy dentures [12]. Actinomyces dominated the denture surface in mature denture biofilms [12, 49]. On the other hand, the presence of Rothia was greater in the 3D printed group than in the conventional group, and this genus was strongly associated with healthy oral conditions [46].

Candida was found in only one patient after 6 weeks of wearing the 3D printed denture. This was not surprising considering that Candida typically appears later in biofilm formation, depending on the thickness of the extracellular polymeric substances [15]. Similar findings were reported by other studies where Candida was also found on dentures of patients with healthy mucosa and those who had been wearing dentures for quite some time without stomatitis [4, 50]. Candida was positively correlated with *Lactobacillus* sp. but negatively correlated with *Fusobacteria* sp [4]. Furthermore, our result found after 6 weeks, *Lactobacillus* sp. levels were low, while Fusobacteria remained in the top ten in abundance, thus supporting previous research [4] on health conditions.

Our findings revealed an increase of LTF and HTN levels following 6 weeks of wearing a newly fabricated denture. Nevertheless, there was no clinical evidence of mucosal inflammation in all patients. This observation could be attributed to the denture being perceived as hygienically maintained. Analysis of bacterial composition of the denture biofilm at week 6 exhibited a more diverse community, suggesting that the biofilm was at a symbiotic stage, hence absence of clinical inflammation.

In addition, no incidence of stomatitis was clinically observed. However, the level of LTF biomarkers was significantly increased in both types of dentures, suggesting an inflammatory response at molecular level. In healthy denture wearers, a few chronic inflammatory cells can be present [51]. Therefore, an increase in LTF level, reflecting an anti-inflammatory reaction, can be expected in both dentures. During the course of the study, patients were instructed to clean their dentures under running tap water without brushing to minimise disruption of the denture biofilm. Without chemical or mechanical brushing, the microbial composition and biomass of undisturbed denture biofilms increased, potentially triggering inflammatory reactions that led to increased LTF level [13].

Increasing biomass of denture biofilm would provide suitable growth condition for Candida. The extracellular polymeric substances (EPSs) in biofilms provide suitable environmental conditions for interactions between Candida and bacteria [15] and nutrients supply for Candida [52]. During week 1 and week 3, the presence of Candida was considered as part of normal oral microbiome and its presence did not cause denture stomatitis. However, by week 6, there was a noticeable increase in denture stomatitis biomarkers in conventional dentures group that could have been triggered by a particular microbe. In contrast, presence of Candida on 3D printed dentures was not related to levels of biomarkers associated with denture stomatitis.

This suggests that the microbiome of the 3D printed denture was not dysbiotic. Our clinical findings suggest that 3D printed dentures may have a potential to reduce the risk of denture stomatitis compared to conventional dentures. Even though, the was an increased in inflammatory response towards 3D printed dentures but there was no antifungal response. Further investigations with larger sample size are warranted to validate these findings.

## Conclusion

The microbial compositions of early biofilm formation were similar between 3D printed and conventional HC denture base resins, however after 6 weeks of denture insertion, mature biofilm on the surface of 3D printed denture demonstrated higher diversity compared to that of HC denture. HTN biomarkers showed presence of Candida in conventional HC denture only. This suggests that 3D printed denture may be a viable alternative to conventional heat-cured resin as it has a potential to delay microbial dysbiosis. However, to date, in view of the conflicting findings, no consensus has been reached on this. Further investigations are needed to better understand the factors affecting the diversity of bacterial composition on 3D-printed dentures in relation to hydrophobicity and surface characteristics of the denture base.

**Clinical implications** 3D printed DBR could serve as an alternative to conventional heat-cured resin because it has a more diverse microbiome and a healthier species composition. It could slow the transition to dysbiosis.

## Electronic supplementary material

Below is the link to the electronic supplementary material.

Supplementary Material 1

Supplementary Material 2

Supplementary Material 3

Supplementary Material 4

Supplementary Material 5

## Data Availability

The raw sequences have been deposited in the NCBI BioProject repository under the accession numbers PRJNA1121639. These SRA records will be accessible via the following links: https://www.ncbi.nlm.nih.gov/bioproject/PRJNA1121639. Other material collected for this research is available upon reasonable request.
