# Supplementary figures and images for: Denture microbiome shift and changes of salivary inflammatory markers following insertion of 3D printed removable partial PMMA denture: a pilot study
Source: BMC Oral Health. 2024 Oct 14;24:1216. doi: 10.1186/s12903-024-05012-z (PMC11476878; doi:10.1186/s12903-024-05012-z)

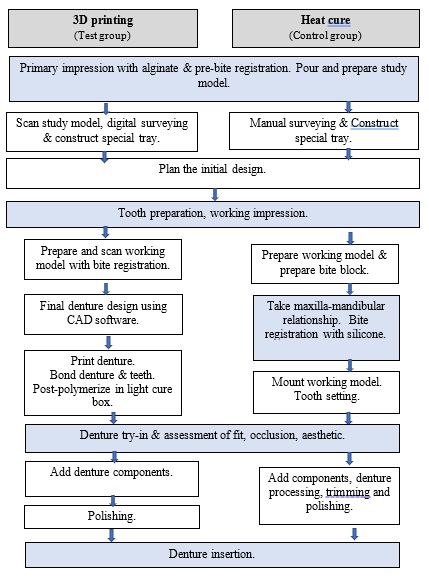

Supplement: Supplementary file 1 — Supplementary Material 1 [file 12903_2024_5012_MOESM1_ESM.jpg]

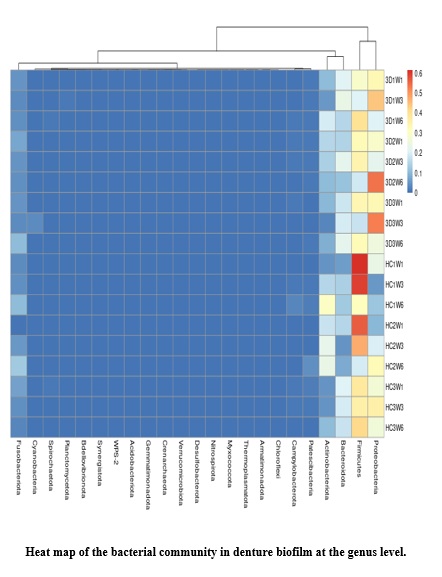

Supplement: Supplementary file 3 — Supplementary Material 3 [file 12903_2024_5012_MOESM3_ESM.jpg]

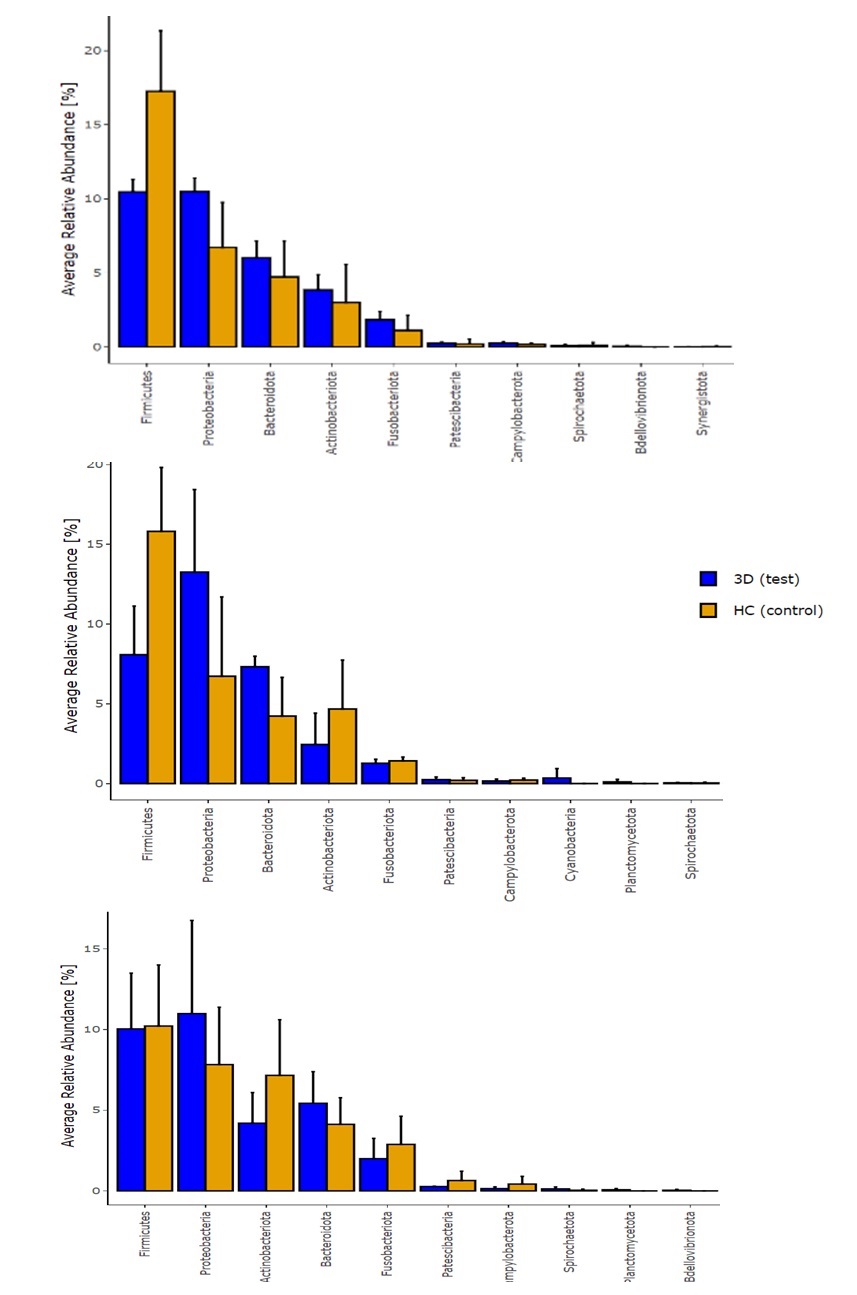

Supplement: Supplementary file 4 — Supplementary Material 4 [file 12903_2024_5012_MOESM4_ESM.jpg]
