# Supplementary material for: Denture microbiome shift and changes of salivary inflammatory markers following insertion of 3D printed removable partial PMMA denture: a pilot study
Source: BMC Oral Health. 2024 Oct 14;24:1216. doi: 10.1186/s12903-024-05012-z (PMC11476878; doi:10.1186/s12903-024-05012-z)
Supplement: Supplementary file 2 — Supplementary Material 2 [file 12903_2024_5012_MOESM2_ESM.docx]

SUPPLEMENTARY 2

Table for supplementary. The overview of sequence result table

| Sample No | Subject number | Sample ID | Treatment time point | Treatment group | Raw data counts | Final read counts (ASV) |
| --- | --- | --- | --- | --- | --- | --- |
| 1 | 1 | 3D1W1 | week-1 | test | 122715 | 98241 |
| 2 | 1 | 3D1W3 | week-3 | test | 140174 | 112658 |
| 3 | 1 | 3D1W6 | week-6 | test | 120856 | 94442 |
| 4 | 2 | 3D2W1 | week-1 | test | 134883 | 106833 |
| 5 | 2 | 3D2W3 | week-3 | test | 143022 | 112318 |
| 6 | 2 | 3D2W6 | week-6 | test | 133129 | 105559 |
| 7 | 3 | 3D3W1 | week-1 | test | 132291 | 105452 |
| 8 | 3 | 3D3W3 | week-3 | test | 221454 | 181829 |
| 9 | 3 | 3D3W6 | week-6 | test | 138997 | 108477 |
| 10 | 4 | HC1W1 | week-1 | control | 131423 | 108219 |
| 11 | 4 | HC1W3 | week-3 | control | 117050 | 98129 |
| 12 | 4 | HC1W6 | week-6 | control | 125759 | 94118 |
| 13 | 5 | HC2W1 | week-1 | control | 120539 | 98970 |
| 14 | 5 | HC2W3 | week-3 | control | 114409 | 99070 |
| 15 | 5 | HC2W6 | week-6 | control | 139831 | 113124 |
| 16 | 6 | HC3W1 | week-1 | control | 105242 | 86960 |
| 17 | 6 | HC3W3 | week-3 | control | 141562 | 114019 |
| 18 | 6 | HC3W6 | week-6 | control | 143773 | 114522 |
