# Supplementary material for: Denture microbiome shift and changes of salivary inflammatory markers following insertion of 3D printed removable partial PMMA denture: a pilot study
Source: BMC Oral Health. 2024 Oct 14;24:1216. doi: 10.1186/s12903-024-05012-z (PMC11476878; doi:10.1186/s12903-024-05012-z)
Supplement: Supplementary file 5 — Supplementary Material 5 [file 12903_2024_5012_MOESM5_ESM.docx]

SUPPLEMENTARY 5

Mean concentration of the LTF and HTN biomarkers from baseline and week 6 after denture insertion.

| **Biomarkers** | **Type of denture** | **Baseline** | **Week 6** | ***p* value** |
| --- | --- | --- | --- | --- |
|  |  | ng/mL | |  |
| **LTF** | 3D-printed | 0.076 | 0.112 | *p*<0.05* |
|  | HC | 0.076 | 0.110 | *p*<0.05* |
| *p* value | | *p>*0.05 | *p>*0.05 |  |
| **HTN** | 3D-printed | 0.159 | 0.204 | *p>*0.05 |
|  | HC | 0.154 | 0.245 | *p*<0.05* |
| *p* value | | *p>*0.05 | *p*<0.05** |  |

^*^ *^p^*^<0.05, Paired t-test; **^ *^p^*^<0.05, Wilcoxon-rank^
